# Phase I/II Clinical Trials Using Gene-Modified Adult Hematopoietic Stem Cells for HIV: Lessons Learnt

**DOI:** 10.4061/2011/393698

**Published:** 2011-06-13

**Authors:** Ronald T. Mitsuyasu, Jerome A. Zack, Janet L. Macpherson, Geoff P. Symonds

**Affiliations:** ^1^Center for Clinical AIDS Research and Education (CARE Center), University of California-Los Angeles, Los Angeles, CA 90035, USA; ^2^Departments of Medicine and of Microbiology, Immunology and Molecular Genetics, University of California-Los Angeles, Los Angeles, CA 90095, USA; ^3^SpanCyte Pty Ltd., B.O. Box 960, Sydney, Leichhardt, NSW 2040, Australia; ^4^Faculty of Medicine and Centre for Applied Medical Research, St. Vincent's Hospital, The University of New South Wales, Kensington, Sydney, NSW 2052, Australia

## Abstract

Gene therapy for individuals infected with HIV has the potential to provide a once-only treatment that will act to reduce viral load, preserve the immune system, and mitigate cumulative toxicities associated with highly active antiretroviral therapy (HAART). The authors have been involved in two clinical trials (phase I and phase II) using gene-modified adult hematopoietic stem cells (HSCs), and these are discussed as prototypic trials within the general field of HSC gene therapy trials for HIV. Taken as a group these trials have shown (i) the safety of both the procedure and the anti-HIV agents themselves and (ii) the feasibility of the approach. They point to the requirement for (i) the ability to transduce and infuse as many as possible gene-containing HSC and/or (ii) high engraftment and *in vivo* expansion of these cells, (iii) potentially increased efficacy of the anti-HIV agent(s) and (iv) automation of the cell processing procedure.

## 1. Introduction

Highly active antiretroviral therapy (HAART) has greatly improved disease management for individuals infected with HIV-1. However, it is often associated with toxicities, adverse interactions with other drugs, and the emergence of viral resistance [1]. The search for an HIV-1 vaccine has been disappointing [2]. Gene-based therapies aim to inhibit HIV replication by the use of an anti-HIV gene expressed intracellularly; such genes include ribozyme, antisense, aptamer, RNAi, zinc finger nuclease, dominant negative protein, fusion inhibitor, intracellular antibody, and viral decoy approaches [3–35]. Some of these genes have been shown to be safe in phase I clinical trials [4, 10, 12, 17, 20, 28–35]. Ribozymes are small catalytic RNA molecules that can be engineered to target specific RNA sequences [4, 10, 19, 20, 24, 26, 27, 31, 32, 35–40]. 

**Figure 1:**
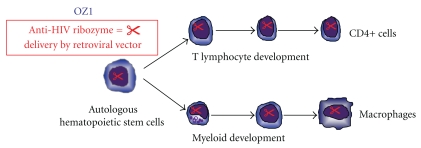
The figure shows the concept of introducing an anti-HIV gene (in this case a ribozyme) into hematopoietic stem cells. As these cells mature and differentiate into T lymphocytes and myeloid cells, the anti-HIV gene is expressed in these cells potentially providing an anti-HIV effect in cells susceptible to HIV.

The gene therapy vector OZ1 (also termed RRz2 in publications) comprises a Moloney murine leukemia virus-based, replication-incompetent gamma retroviral vector (LNL6) containing a gene that encodes a ribozyme targeting the overlapping *vpr *and *tat* reading frames of HIV-1 [4, 32, 36–39]. OZ1 has been shown to inhibit the replication of laboratory and clinical isolates of HIV-1 *in vitro* [36–39]. Resistance mutations in the region of HIV-1 targeted by OZ1 were not observed in long-term cell culture [10, 27, 37, 39]. The concept tested in the two clinical trials conducted by the present investigators and colleagues (phase I and phase II) was that, OZ1-transduced CD34+ HSC would engraft, divide, and differentiate *in vivo* to produce a pool of mature myeloid and lymphoid cells protected from productive HIV-1 replication and, in the case of the phase II trial, that this protection could be measured by differences in plasma HIV-1 RNA levels in the absence of antiretroviral therapy [32]. This concept is shown pictorially in Figure 1. In both these prototypic trials, autologous CD34+ HSC were transduced and administered without the subject undergoing myeloablation or any form of bone marrow conditioning.

## 2. Prototypic Phase I Trial Design

**Figure 2:**
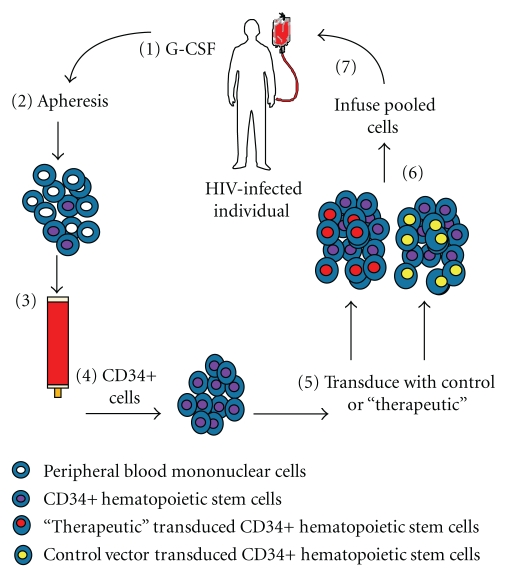
The figure shows the prototypic phase I trial design in which CD34+ HSC were obtained from post G-CSF apheresis product that is CD34+ selected. The CD34+ cells were then transduced with either control or OZ1-containing vector and both transduced populations (control and OZ1) mixed and infused into the individual. (1) Subjects are injected with a course of G-CSF to mobilize CD34+ HSC from the bone marrow to the peripheral blood. (2) Apheresis product is obtained. (3) The mononuclear cell fraction is applied to a CD34+ isolation system. (4) CD34+ cells are obtained. (5) These are transduced with either control or OZ1-containing vector (50% of each) to obtain (6) A mixed population of control and OZ1 transduced cells. (7) This mixed population is infused back into the individual.

The prototypic phase I clinical trial [4] was conducted using CD34+ HSC to assess the safety and feasibility of *ex vivo* transduction and reinfusion of autologous OZ1-transduced cells. The protocol involved injection of the subjects with G-CSF to mobilize HSC into the peripheral blood, collection of the mononuclear cell fraction by apheresis, selection of the CD34+ population, culture of these cells *in vitro* with cytokines, their transduction with control (LNL6) or therapeutic (OZ1) vectors, and finally cell harvest and infusion (see Figure 2). This trial demonstrated that the approach was safe and technically feasible and that concurrent administration of antiretroviral therapy did not inhibit stem cell mobilization or the ability to transduce HSC *in vitro*. There were no serious adverse events related to the gene transfer process or the gene transfer product during the study period or the subsequent long-term safety followup. Detection of the gene in peripheral blood cells and bone marrow cells was found in some patients to be present out to 3 years after a single infusion of these cells [4].

## 3. Prototypic Phase II Trial Design

**Figure 3:**
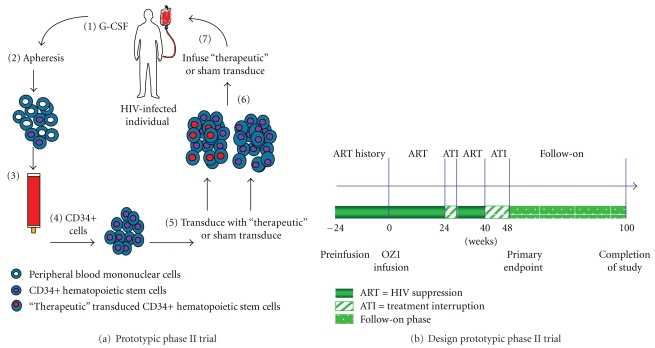
(a) The figure shows the prototypic phase II trial design in which CD34+ HSC are obtained from apheresis product that is CD34+ selected. The cells were then transduced with either sham (medium only) or OZ1-containing vector and that population (control or OZ1) infused into the individual. (1) Subjects are injected with a course of G-CSF to mobilize CD34+ HSC from the bone marrow to the peripheral blood. (2) Apheresis product is obtained. (3) The mononuclear cell fraction is applied to a CD34+ isolation system. (4) CD34+ cells are obtained. (5) These are sham transduced (medium alone) or transduced with OZ1 to obtain (6) a control population or OZ1 tranduced cells. (7) This population is infused back into the individual. (b) The figure shows the schedule for the phase II clinical trial. ART, antiretroviral therapy; ATI: analytic treatment interruption. The primary endpoint was viral load at weeks 47/48. Other end-points were area under the viral load curve weeks 40–48 and 40–100.

In this randomized trial [32], subjects (1 : 1 randomization) were infused with either gene or sham medium only transduced autologous CD34+ HSC. The protocol of cell selection, culture, and transduction was based on the phase I trial, with some modifications. In addition (unlike the phase I trial), the protocol included two antiretroviral treatment interruptions (ATIs) to provide positive selective pressure for OZ1-protected cells, and as a read-out for the effectiveness of these protected cells in preventing further HIV replication *in vivo* (see Figures 3(a) and 3(b)). The impact of OZ1 on plasma HIV-1 viral load was assessed at the end of the second eight-week ATI (the primary endpoint). Secondary endpoints of quantitative marking (presence of gene) and expression (active RNA form) of OZ1, time-weighted area under the curve for viral load (TWAUC), CD4+ T lymphocyte count in absolute and percentage of T lymphocytes (CD4%), presence of HIV-1 proviral DNA, and thymic function (T cell receptor excision circles, TREC) were assessed at the primary endpoint (weeks 47/48) and to week 100. The OZ1 treatment group participants are now enrolled in a long-term safety followup protocol.

## 4. Regulatory Process

For the prototypic trials, approval was obtained from the relevant Institutional Review Boards/Human Research Ethics Committee as well as the Recombinant DNA Advisory Committee (RAC), the Center for Biologics Evaluation and Research (CBER), the United States Food and Drug Administration (FDA), and the Australian Therapeutic Goods Administration (TGA). Safety parameters were assessed in accordance with RAC, CBER, FDA, and TGA recommendations [41]. The majority of the other trials followed similar routes of approval, predominantly in the USA (see Table 1).

## 5. Cell Processing

In both the prototypic phase I and phase II clinical trials the cell processing involved the elements as depicted in Figures 2 and 3(a). Apheresis was performed using the COBE Spectra Apheresis System (Gambro) and the mononuclear cell fraction subjected to CD34+ cell selection using the CEPRATE SC Stem Cell Concentration System (CellPro Inc) (initial subjects in phase I trial) [4] or the Isolex 300i Cell Selection System (Nexell Therapeutics) (all other subjects phase I and II trials) [4, 32].

### 5.1. Phase I Trial [4]

The CD34+ selected cell population was placed into tissue culture with Iscove's Modified Dulbecco's Medium (IMDM) containing the cytokines stem cell factor (SCF) and megakaryocyte growth and development factor (MGDF) at a concentration of 50 ng/mL and 100 ng/mL, respectively (Amgen Inc). Cells were split into two equal aliquots and transduced on day 2 with either an LNL6 control or the OZ1 retroviral vectors (Figure 2). In the 7 latter subjects, transduction was improved by the use of RetroNectin resulting in a range of transduction from 7 to 57%. Cells were collected on day 3, and after release testing, the two separately transduced HSC aliquots were combined and infused into the subject from whom they were obtained. The number of CD34+ HSC infused was in the range of 1–10 × 10^6^/kg.

### 5.2. Phase II Trial [32]

Similar to the phase I trial, the CD34+ selected cell population was placed into tissue culture with Iscove's Modified Dulbecco's Medium (IMDM) containing the cytokines stem cell factor (SCF) (Amgen) and megakaryocyte growth and development factor (MGDF) (Takara) at a concentration of 50 ng/mL and 100 ng/mL, respectively. However in this case, subjects were randomized to receive either sham (medium alone) or OZ1-transduced CD34+ HSC (Figure 3(a)). Both treatment groups received an equivalent dose of viable CD34+ cells/kg (9 × 10^6^/kg) and the infused cell product in the OZ1 treatment group contained a mean of 54.0% OZ1-containing cells. Participants in the OZ1 group are now enrolled in a separate long-term follow-up protocol which will continue for at least 15 years. To date, the longest follow-up period from the time of infusion of the first participant is 6.5 years [32].

## 6. OZ1 Gene Marking (DNA) and OZ1 Expression (RNA)

In the prototypic phase I trial [4], hematopoietic progeny cells containing either the LNL6 vector or OZ1 (derived from the gene-modified CD34+ HSC) could be separately monitored in the subjects, and it was estimated that 0.001–0.01% gene-containing progeny cells were present in the subjects' peripheral blood. Transgene expression was shown in peripheral blood mononuclear cells and T-cell subsets including naive (CD45RA+CD62L+) T lymphocytes. In the phase II trial [32], OZ1 gene marking (DNA) and expression (RNA) were also assessed, with the degree of gene marking in peripheral blood mononuclear cells higher than in the phase I trial at 0.01–0.38%.

## 7. Efficacy in Phase II Trial [32]

Statistical evidence of antiretroviral efficacy found in the phase II trial was as follows (OZ1 versus control):

1. Plasma viral load;
  - greater number of subjects with a plasma viral load of less than 4 log_10_ copies/mL at weeks 47/48 (15/32 versus 5/33) (*P* = .009);
  - longer median time (36 versus 24 days) to reach 4 log_10_ copies/mL viral load during the analytic treatment interruption.
2. The time-weighted area under the log viral load curve (TWAUC) was statistically lower in the OZ1 group (weeks 40–48; median difference −0.34 log_10_ copies/ml/day, *P* = .024 and weeks 40–100; median difference −0.37 log_10_ copies/ml/day, *P* = .034).
3. The number of participants with a TWAUC in the lowest quartile during weeks 40–100 was statistically greater in the OZ1 group (OZ1 *n* = 12; 37.5%, Control *n* = 5; 15.2%, *P* = .04).
4. Median plasma viral load in the OZ1 participants who continued to display OZ1 expression in PBMC at any time point beyond week 48 (3.81 log_10_ 95% CI; median 3.18–4.23; *n* = 15) was significantly lower than that in the control participants (4.58 log_10_ 95% CI; median 4.31–4.83; *n* = 33) (*P* = .003). The median TWAUC from weeks 40–48 in these OZ1 participants (3.44 log_10_ copies/ml/day, *n* = 15) was significantly lower than that in the control participants (3.93 log_10_ copies/ml/day, *n* = 33) (*P* = .03) as was the median TWAUC from weeks 40–100 (3.97 log_10_ copies/ml/day, *n* = 15) in comparison to the control group (4.53 log_10_ copies/ml/day, *n* = 33) (*P* = .005).

Other trends were as follows.

1. Mean plasma viral load was lower in the OZ1 group at 47/48 week (primary endpoint).
2. During the analytic treatment interruption, 17 (45%) participants in the OZ1 group reinitiated HAART compared to 22 (61%) control participants.
3. Median time to reinitiate antiretroviral therapy during analytic treatment interruption was 29.4 weeks in the control group (*n* = 22, 61%) and for the OZ1 group was greater than 60 weeks.
4. CD4+ T lymphocyte numbers and % CD4 were higher, and CD8+ T lymphocyte numbers lower, in the OZ1 group.

## 8. Safety Evaluations in Prototypic Trials in addition to SAEs

In both prototypic phase I and II clinical trials no replication-competent retrovirus was detected at any time point. In both trials no clonal expansion of hematopoietic cells or other event suggestive of insertional mutagenesis was observed; predominant integration site analysis was only required in the phase II trial. The OZ1 target sequence in the HIV-1 plasma RNA was assessed over time, and there was no modification at the ribozyme recognition site to prevent cleavage, or to drive the evolution of resistant virus.

## 9. Summary of Other Stem Cell Trials for HIV/AIDS

Other trials that have been conducted in stem cells for the indication of HIV/AIDS are summarized in Table 1.

## 10. Discussion

Gene therapy for HIV-positive individuals has the potential to provide a once-only treatment that reduces viral load, preserves the immune system, and avoids cumulative toxicities associated with HAART. The two prototypic clinical trials (phase I and phase II) described used *tat/vpr*-specific anti-HIV ribozyme (OZ1) gene-modified autologous HSC. In these two trials, 10 subjects [4] and 74 subjects [32] were treated, respectively. 

These and trials from other investigators have shown the safety of the procedure and the anti-HIV agents themselves as well as the feasibility of the approach in which autologous CD34+ HSC are taken from the subject, genetically manipulated and given back to the subject. In addition, the phase II trial demonstrated a significant biologic effect. 

The rationale for the prototypic trials was to demonstrate a “HAART-sparing” function of the therapy, that is, the ability to decrease, partially or totally, the need for HAART. While an antiretroviral effect was seen in the phase II study, it was relatively modest.

Taken together with the other stem cell trials summarised, areas for focus appear to be (i) maximizing the number of gene-containing HSC transduced and infused and/or (ii) maximizing the engraftment, proliferation, and differentiation of these gene-modified cells, possibly by incorporating partial myeloablation; (iii) potentially increasing the effectiveness of the anti-HIV gene(s) used and (iv) automation of the cell processing procedure. Each of these points are discussed here.

### 10.1. Number of Gene-Containing HSC and Subsequent Engraftment

In both prototypic clinical trials, a dose of 10 × 10^6^ CD34+ HSC/kg could be achieved. Improvements in mobilisation, CD34+ cell collection, and transduction compared to the phase I study [4] resulted in a mean transduced CD34+ cell dose of 5 × 10^6^ cells/kg and an increase (approximate 2 log_10_) in the frequency of OZ1-containing cells in the peripheral blood. In both trials (reflected in results from other investigator trials), the frequency of gene-containing cells in the peripheral blood decreased over time indicating a need to potentially take care of one or several of the following in relation to the HSC: (i) maximizing the number of gene-modified cells, (ii) maximizing the degree of engraftment, (iii) increasing the degree of proliferation and differentiation, (iv) infusing on more than one occasion.

It is relevant that, in each trial, transduced HSC infusion resulted in gene marking in peripheral blood of only a maximum of 0.01% (phase I trial) [4] and 0.38% (phase II trial) [32]. It is significant that mathematical modeling undertaken prior to the phase II trial [42] predicted that, during the analytic treatment interruption, OZ1 recipients would experience an initial increase in HIV-1 viral load followed by the establishment of a lower set point. This is indeed the result that was seen in the phase II trial itself [32]. The model predicted that the establishment of OZ1 CD34+ cells in the bone marrow at approximately 5–10% of total CD34+ cell population could reduce viral load by 0.5 log_10_ in one year. In this phase II study, bone marrow aspiration was not performed, and hence the percentage engraftment is not known. Based on the frequency of cells containing OZ1 in the Phase II trial (0.01% to 0.38%) in the peripheral blood, it can be inferred that engraftment was substantially lower than 5–10%. Previous studies have also shown that, in the absence of strong selective pressure, peripheral blood reconstitution with gene-containing cells is limited [43–46]. Given the engraftment and gene expression results in the phase II study [32], the antiretroviral effect of OZ1 was greater than predicted by the modelling. Candidate mechanisms for this additional effect include an impact of OZ1 on cell-to-cell transmission in the lymphatic system, compartmentalisation such that the number of OZ1-containing cells in the peripheral blood is not representative of the survival of OZ1-containing cells in sequestered foci (e.g., the bone marrow and various lymphatic tissues, such as the GI lymphoid pool), and perhaps protection of particular cell subpopulations such as antigen-presenting cells (including dendritic cells and macrophages) or HIV-specific CD4+ T lymphocytes. A recent report [47] suggests that early hematopoietic progenitor cells in the bone marrow might be infected with HIV. It is possible that OZ1 in our phase II study targeted this reservoir, and this could allow for a larger biologic effect than that predicted by the mathematical model. 

It is also of high interest to explore ways to expand and ensure efficient engraftment *in vivo *by use of factors such as SDF-1 and growth factors/cytokines/differentiation factors. Therefore, by increasing the transduction efficiency, facilitating engraftment of these reinfused cells, and potentially using multiple infusions of transduced cells over time, it may be possible to increase the number of these gene-protected cells to see an even greater antiretroviral effect in the future.

### 10.2. Increased Efficacy of the Anti-HIV Agent and Potential Use in Different Patient Populations

In both clinical trials, the gene transfer agent was a retroviral vector delivering a *tat/vpr*-specific anti-HIV ribozyme. While effective at inhibiting HIV in tissue culture systems, it may be that this agent will need to be used in combination with other anti-HIV genes or other agents to produce a greater therapeutic effect and to overcome potential resistance. The use of multiple agents targeting several sites in the viral replication cycle is now a well-established concept in anti-HIV therapy. 

In the phase I and phase II trials described here, individuals were recruited who were on a relatively (phase I) or fully (phase II) effective HAART regimen. Future evaluation may include individuals prior to HAART initiation in whom viral load (and hence selective pressure for gene-containing cells) will be higher without the need for treatment interruptions. Other potential patient groups consist of individuals who are multidrug resistant or have AIDS and have no other options for therapy. It should be possible to trial this therapy on these varying patient populations and eventually there may be an indication that includes all HIV positive individuals.

### 10.3. Automation of Cell Processing

The cell processing procedures used in the phase I and II trials were multi-step, requiring skilled operators and a variety of equipment/devices. Automation of this process, using a completely closed system, would be highly desirable to (i) reduce operator time and effort and the potential for errors, (ii) potentially increase reproducibility and reliability of the approach, and (iii) avoid the risk of contamination. Such an automated and closed system would be highly desirable for high patient access and reduced cost of cell processing.

### 10.4. Areas for Maximizing Effect

These can be summarized as

- number of gene-containing cells in the peripheral blood and lymphatic tissue;
- apparent efficacy of anti-HIV agent;
- improve relatively laborious cell processing.

### 10.5. Positive Outcomes

Notwithstanding the areas for maximizing effect, the prototypic studies indicate that OZ1 cell-delivered gene therapy is safe and has antiretroviral activity, albeit modest. Our phase II trial also showed that a large number of subjects (74) can be treated similarly at several (3) distinct clinical sites, indicating that this type of approach can be scaledup to treat substantial numbers of patients, if personnel at the clinical site are properly trained in cell processing and delivery. These trials show the potential of the gene therapy approach for the treatment of HIV-1 and represent a major advance in the field.

## Figures and Tables

**Table 1:** 

Target/mechanism of action	Construct	Results
Rev [30]	“Humanized” dominant-negative REV protein (huM10) and nontranslated marker gene (FX) as an internal control in retroviral vector	Gene marking in first months, then low or undetectable except in one patient when viral load increased. No serious adverse events.
RRE decoy [12, 33]	Retroviral-mediated transfer of an RRE decoy gene into bone marrow CD34+ cells	No adverse effects. 2 subjects' cells detected containing both the RRE and LN vectors on the day after cell infusion. All subsequent samples negative for the L-RRE-neo vector. Cells containing the control LN vector detected up to 330 days.
Rev/tat ribozyme [34]	Tat and tat/rev ribozyme in autologous CD34+ cells and empty vector backbone in two patient groups with and without ablation	Trial 1: 3/5 patients showed low-frequency marking of PBMC with ribozyme and vector backbone. Trial 2: gene marked cells detected after infusion and to 1 year and RNA expression detected.
Tat/vpr ribozyme [4]	Phase I study: Moloney murine leukemia retroviral vector encoding a ribozyme versus control LNL6 vector in CD34+ HPSC	*De novo* production of myeloid and lymphoid cells. Degree of persistence of gene-containing cells dependent on transduced cell dose.
Tat/vpr ribozyme [32]	Phase II study: Moloney murine leukemia virus-based, replication-incompetent gamma retroviral vector with gene encoding a ribozyme vs placebo in CD34+ cells	No significant difference mean plasma viral load at primary endpoint but lower TWAUC and other indicators of biologic effect. No safety concerns.
Tat/rev, CCR5, TAR decoy [35]	Tat/rev short hairpin RNA, TAR decoy, and CCR5 ribozyme expressed from a self-inactivating lentiviral vector transduced in CD34+ cells, along with standard unmanipulated HPCs in 4 patients with HIV and non-Hodgkin's lymphoma	Engraftment by 11 days. Low levels of gene marking observed up to 24 months.
